# Meaning of home and health dynamics among younger older people in Sweden

**DOI:** 10.1007/s10433-019-00501-5

**Published:** 2019-01-24

**Authors:** Maya Kylén, Charlotte Löfqvist, Maria Haak, Susanne Iwarsson

**Affiliations:** Department of Health Sciences, Lund University, P.O. Box 157, SE-221 00 Lund, Sweden

**Keywords:** Perceived aspects of home, Ageing, Third age, Qualitative study, Housing

## Abstract

Research has shown that positive evaluations of home are important for very old people’s health, well-being and independence in daily life. The rationale for the present study derives from our survey study findings, confirming such associations also in a younger cohort (*N* = 371). The purpose of this study was to further increase the understanding of the dynamics of meaning of home and health among community-living healthy younger older people, in the present and in a projected future. Data were collected through semi-structured interviews with 13 persons aged 67–70 years living in ordinary housing in Sweden, followed by a qualitative content analysis. Findings suggest that the home becomes progressively important after retirement. Not only the immediate home environment but also local neighbourhoods influence perceptions about home. Home brings emotional and social benefits but also worries about how to cope with complex home ambivalence when reflecting upon future housing arrangements. The findings suggest that it is important to consider the role of perceived aspects of home for health and well-being in early phases of the ageing process. The findings could be used to raise awareness among policymakers, housing authorities and professionals involved in housing-related counselling.

## Introduction

The share of older people in the population is increasing worldwide, and this phenomenon has been noted to have consequences for housing provision, in Sweden (SOU 2015:85) as well as internationally (Walker 2011). This includes an increasing demand for accessible housing (United Nations 2015), stressing the need not only to build new dwellings but also to adapt the current housing stock to fulfil older people’s needs.

The majority of older people wish to age in place, meaning to maintain long-term residence which in turn improves health and well-being (Gustafson 2014). The current housing policies in many countries favour support for people to age at home for as long as possible, regardless of frailty or need for services and care (Iecovich 2014). However, researchers, policymakers and the general public are now questioning these predominating norms. Older people do not have shared needs and wishes (Weicht 2013) and the current policy approach might hinder senior citizens from seeking more suitable housing alternatives. Today’s older generation is different from their predecessors in terms of education, work opportunities, gender roles and perceptions about ageing (Pruchno 2012). Even though it is well established that the home is the major context for ageing (Rowles and Bernard 2013) and previous research has shown that perceived aspects of home are related to health in very old age (Iwarsson et al. 2016), research exploring such dynamics in younger cohorts remains scarce. In order to inform policymakers and authorities about the best possible housing options as people age, research confirming or contrasting such findings in younger cohorts with different levels of competencies and life experiences is warranted.

Home is a multi-layered concept different from that of housing. A house is a space and a building, while a home is a place filled with meanings and personal experiences, over time transforming space (house) into place (home) (Rowles and Bernard 2013). The home develops to be a part of an individual’s life and identity (Dahlin-Ivanoff et al. 2007). This is especially true in the later stages of life when the home becomes the key spatial centre, serving to preserve independence in the face of declining function (Rowles and Bernard 2013). We use concepts derived from a model of perceived housing in later life to address the personal connection and symbolic representations that make a house a home (Oswald et al. 2006). This model includes meaning of home, usability, housing-related control and housing satisfaction. Meaning of home refers to phenomena concerned with being in place, and subjective meanings linked to home. Usability highlights to what extent the home can be used to perform necessary and favoured activities. Housing-related control has its origin in person–environment fit processes (Oswald et al. 2003) and psychological theories on perceived control (Heckhausen and Schulz 1995) elucidating to what extent the individual feels in control at home (Oswald et al. 2006). Supported by survey studies, these perceived aspects of home are important for well-being and independence among people aged 80–89 (Iwarsson et al. 2016; Oswald et al. 2007) and for symptoms and psychological well-being among people aged 67–70 years (Haak et al. 2015; Kylén et al. 2017). Recently, Ekström et al. (2016) compared perceived aspects of home among people aged 81–87 with people aged 67–69 living in ordinary housing in Sweden. Several differences between these cohorts were identified; for instance, in comparison with the very old cohort, the younger appreciated the social aspects of home more. This was, however, not found by Oswald et al. (2010) in Germany, showing that having social partners close to home and social neighbourhood quality had no effect on life satisfaction among people aged 65–79 years. This divergence in findings might reflect different study contexts or that quantitative assessments used to capture perceived aspects of home fail to account for the multidimensional factors of person–environment processes (Oswald and Kaspar 2012).

Several qualitative studies have examined what the home means in old age, but the majority focused on very old people (80+), or older women. Findings show that home can be defined as a resource; an attachment; the precariousness of maintaining and sustaining home, and a cultural expectation (Barry et al. 2017). Home is important in terms of security and freedom (Dahlin-Ivanoff et al. 2007) and for health (Fänge and Dahlin-Ivanoff 2009). Some studies focused on housing needs (e.g. Bigonnesse et al. 2014) or the meaning of home (e.g. Board 2014) among younger older people; however, studies exploring the broader picture of perceived aspects of home and health in this phase of ageing remain rare. To foster and develop efficient health promotion strategies that support active and healthy ageing, there is a need to address this knowledge gap.

The rationale for the present study derives from findings in our previous survey studies showing associations between perceived aspects of home and health among 371 people aged 67–70 years (Haak et al. 2015; Kylén et al. 2017). In order to further deepen the understanding of these phenomena, the overarching aim of this study was to explore the dynamics of perceived aspects of home and health among younger older people. We explored their experiences of home in terms of meaning, usability and housing satisfaction, as well as the importance of being in control over the housing situation, in the present and in a projected future.

## Methods

A purposeful sampling was used to identify and select participants (Patton 2002). Striving for various experiences of home, we selected potential participants in a step-wise process based on criteria such as sex, geographical area, type of housing and length of time lived in the present home. Eligible participants were 67–70 years old living in ordinary housing in southern Sweden, able to read and speak Swedish and willing to share experiences with a researcher in an interview.

Presidents of the two largest national senior citizens’ organizations were contacted and asked to identify contact persons within three local organizations mirroring a diversity in size and type of geographical areas (rural/urban). Six persons were suggested and contacted by the first author (MK) who provided verbal and written information about the study. The contact persons were asked to identify five members each (*N* = 30) whereof 23 accepted to be contacted by a researcher. When contacted, one person had changed his/her mind and four could repeatedly not be reached by phone. Striving for diversity, the potential participants were asked questions regarding their housing situation which included cohabiting/living alone, one-family/multi-family house and number of years in the present dwelling. The final selection of 13 participants was made considering these variables.

### Ethics

The study was conducted in accordance with the Helsinki Declaration and approved by the Regional Ethical Review Board in Lund, Sweden (2016/662). Written informed consent was obtained from all participants. Confidentiality was ensured, and participants were informed that they, without reason, could withdraw from the study if and whenever they wished.

### Participants

The participants perceived their health as good or very good, had few functional limitations and were autonomous in everyday life. All participants (8 women, 5 men) had retired around the age of 65, were cohabiting, community-dwelling and aged 67–70 years (median = 69 years). Six participants were living in one-family houses and seven in apartments in multi-family housing. More than half had lived in their current dwelling for > 15 years (range = 2–41). See Table 1.

**Table 1:** Characteristics of participants, *N* = 13

	All	Men	Women
Sex	13	5	8
*Age*			
67	1		1
68	2	1	1
69	5	1	4
70	5	3	2
*Housing type*			
Apartment	7	3	4
House	6	2	4
*Years in the present home*			
≤ 5	3	1	2
6–16	4	2	2
17–24	2	1	1
25–38	2	1	1
39–41	2		2
*Education*			
Elementary school	6	3	3
Secondary school	4	1	3
University	3	1	2
*Self*-*rated health*			
Poor	0		
Moderate	1		1
Good	7	3	4
Very good	5	2	3
Excellent	0		
Depressive symptoms^a^			
≥ 5	1		1

^a^Geriatric Depression Scale (GDS) ≥ 5 indicates depressive symptoms (Almeida and Almeida 1999)

### Data collection

We developed a semi-structured interview guide inspired by the four-domain model of perceived housing (Oswald et al. 2006) as well as previous knowledge on home and health dynamics among people aged 67–70 years (Haak et al. 2015; Kylén et al. 2017). The interview guide was tested, refined and optimized based on two pilot interviews with people representing the target group (not in the sample).

MK conducted the interviews at home visits during three winter months. Open-ended questions such as “If you think about your home and what it means to you, what are your direct thoughts?” encouraged the participants to talk and reflect about their experiences of home. Follow-up questions such as “what is it that makes you feel at home, activities in the home” were asked. Verbal and nonverbal probing techniques (Patton 2002) generated further explanations. At the end of the interview, the participant had the possibility to add complementary information. For descriptive purposes, each participant answered questions regarding perceived health (five response alternatives from 1 poor to 5 excellent) (Ware 1992) and depressive symptoms dichotomously assessed with the 15-item Geriatric Depression Scale (GDS) (Almeida and Almeida 1999). The interviews lasted 45–70 min and were audio-recorded and transcribed verbatim.

### Data analysis

Inspired by Elo and Kyngäs (2008) and Hsieh and Shannon (2005), we utilized an integrated deductive and inductive analysis approach. To guide our initial interpretation, we used concepts from the four-domain model (Oswald et al. 2006) in directed content analysis (Hsieh and Shannon 2005), followed by inductive content analysis (Elo and Kyngäs 2008). In order to make sense of the data and obtain a sense of the whole, MK read all transcripts several times. A categorization matrix (Elo and Kyngäs 2008) including the concepts meaning of home, usability and housing-related control was created. Together with empirical results from our previous studies (Haak et al. 2015; Kylén et al. 2017), these concepts were used as a lens in the first stages of analysis. A description of what could be covered by each concept was created, and data matching the predetermined categories were placed into the matrix. Applying an inductive approach, data that did not fit the categorization were organized in additional categories and subcategories (Hsieh and Shannon 2005).

Next, the content of the categories and subcategories was inductively analysed and discussed by MK and the second author (CL). It was evident that the theoretically proposed concepts did not fully cover the complexity of the data. In theory, each concept is defined and clearly separated from the other. In practice, perceptions as told by the participants were far more multifaceted and intertwined. Thus, the whole set of data was once again coded, resulting in five categories with subcategories.

In order to enhance trustworthiness, peer debriefing meetings with all co-authors were held throughout the analysis process (Lincoln and Guba 1985). During these meetings, co-authors (MH, SI) acted as critical discussion partners. All feedback was considered and integrated. The analysis was aided by the NVivo 11 software (Edhlund and McDougall 2010).

## Findings

The spatial margins and the meaning people attach to their home emerged as individual and dynamic. Health was described in terms of feeling good at home, being attached and performing enjoyable activities. Home was an important social setting, especially in maintaining a sense of purpose after retirement. Five categories emerged, some with subcategories (see Table 2 for an overview).

**Table 2:** Overview of categories and subcategories

Category	Subcategories	Description
Contextualizing attachment to home		The findings revealed that attachment to home is not only influenced by personal and social experiences but also by the experience of places beyond home that contribute to health. Thus, during this early phase of the ageing process, home was described as more than one place and the interrelationship between those places is important for health
Home as a reference point, a place of departure and return	Home as a base	This category covers the importance of the spatial location of the home. It pertains to aspects such as distance to other places, location or geographical appearance linked to the experience of home and well-being
	Living in a well-designed placed geared to my individual needs	
Social engagement deepens the meaning of home	Social interactions and roles	Social interactions were important for the experience of home and expressed to be reliant on places to meet. The category reflects that the home experience is shaped and deepened through interaction with significant others
	Organized social activities and learning new skills	
Transition in perceptions and behavioural meaning of home post-retirement		This category denotes that the experience of home is intertwined with performing everyday activities. More important, behavioural meaning-related aspects of home are dynamic and change over time
Coping with complex home ambivalence when reflecting on their future housing arrangements		This category reflects that planning for the future is a multifaceted and highly individual process characterized by complex home ambivalence. The mixed feelings voiced by the participants are not only due to health issues but also to identity and societal norms. The diversity of older adults’ reasoning, their wishes and specific needs concerning their future housing arrangements was prominent

### Contextualizing attachment to home

The findings revealed that how the home is perceived is influenced by the experience of places beyond home. Thus, during the early phase of the ageing process, home was described as more than one place and the interrelationship between those places emerged as important for health.

The participants described home in emotional terms as a warm and relaxing place. On the one hand, home was narrowly defined and only included the immediate dwelling. On the other hand, the home was described in a wider context including places in the close neighbourhood. When retirement made it possible to spend more time in a summer cottage or in the courtyard, these places became increasingly important. As said by one participant, a former builder:

Now when I'm retired I spend much more time in our summer cottage, before (retirement) we were only there during the holidays.

The possibility to use these extended home environments for recreation was highly appreciated, and the experiences of being in these places mediated the attachment to home.

Home was also expressed as a place for continuity. Objects and furniture in the home were carefully chosen and created a personal atmosphere. Many of the participants described feeling at home, surrounded by emotionally meaningful objects, as being closely related to well-being. This is illustrated by a 69-year-old woman, living in a flat in a small municipality, reflected on her experience of home as follows:

It is cozy and…I feel good when I come home […] it is familiar in a way, I see my belongings that I had for a long time and I come home to…yes it feels warm, but I can't explain why […] it brings certainty, that’s what it is.

Inherited objects such as paintings and old furniture were treasured. Although often linked to memories and kept as a reminder of the past, it was not a necessity to feel emotionally devoted to these types of objects. It could be more important that things around the home were functioning and usable.

### Home as a reference point, a place of departure and return

Distance to other places, location or geographical appearance was linked to the experience of home and well-being. The findings are presented in two subcategories.

One woman living in a central apartment since 16 years together with her partner, expressed that her home was the solid foundation in life, a place to which she always returns. Mirroring such reflections, together with aspects of the spatial location in the neighbourhood, is captured in the subcategory “Home as a base”. Several of the participants perceived it as important to be within walking distance from shops and services. Such central location was convenient and supported independence and fostered a sense of control. For example, a 70-year-old man, living on the edge of a semi-urban town, said:

…we have lived further away (from town) before, but then we were young and healthy… (laughing)…now it is ideal for us to have shops and public transport so close.

The location of the home was also important for those who had a summer house to visit. It had to be easy to travel from one home to another. The neighbourhood aesthetics were perceived as essential for well-being. Aesthetic features such as green parks or the ocean encouraged the participants to do their daily exercise.

The subcategory “Living in a well-designed place geared to individual needs” was related to the physical attributes inside the home. The home was made more usable through equipment chosen to fulfil specific needs, expressed through statements like *“When we bought a new bed we chose one that´s adjustable”* or *“Our new TV is on wheels so it´s easy to move around”*. Independence was supported through renovations. For example, one woman adapted her kitchen to suit her short stature and chose telescopic shelves so that she could reach.

### Social engagement deepens the meaning of home

Social engagement was described as important for health and expressed to be dependent on places to meet. This category reflects that perceptions of home are shaped and deepened through engaging in social activities with significant others. The findings are presented in two subcategories.

The participants expressed that home was an important social setting, not only for spending time with family and friends but also for maintaining a sense of purpose after retirement. The subcategory “Social interactions and roles” was thus related to social interactions that were driven by a desire to feel valued and doing something purposeful. For example, as a way to maintain roles and professional skills, the participant who was a former social worker helped her frail neighbour with daily chores, and the retired carpenter took pride in assisting others with renovations. These interactions were driven by internal factors and expressed by the participants as *“Feeling valued”* or *“Doing something purposeful”*.

The participants emphasized the social contact and mutual help they had from neighbours. The communication between neighbours was important in terms of chitchat, and knowing the neighbours created a sense of security. Helping each other was a matter of course and a strategy to secure a network in case of personal needs. Thinking about the years to come, one woman who had lived in the same house for 38 years said: *“One day we will need help, if we help others they will help us”*. Helping each other was mentioned as important by all, however, for those with poorer health or caring for a spouse the social aspects were of significant value:

Yes, it means a lot… it feels good to have someone, if anything happens I can just call…they know that my husband is ill so they take extra care of me.

Social interactions with peers in the same age were important in terms of feeling included and being able to exchange thoughts. One woman had moved from an urban age-integrated environment to a small forest community where the majority of residents were seniors. She described this move as *“Coming home”*. A man living in a similar environment since 21 years said that he liked to live close to peers as they have *“The same values about life”.*

Experiences of positive outcomes from getting involved in social activities emerged in the subcategory “Organized social activities and learning new skills”. Participants living in condominium apartments spoke highly about the benefits of getting involved in arranged activities (e.g. ritual seasonal celebrations and festivities). Engaging in such activities prevented loneliness and was expressed as *“A good way to meet people”* and as *“Something to look forward to”*.

Getting involved also meant learning new skills and inspiring each other. One healthy 70-year-old woman, living in the countryside, revealed that the close neighbours applied for an EU grant together to build a community hall. *“We thought that we needed a place to meet”* she said, now used frequently for language and dance courses. Another example, one man who had worked as a bus driver, being used to meet a lot of people on a daily basis, revealed that he had enlisted two of his senior neighbours to participate in gymnastics. He valued these social gatherings and said: *“Afterwards we sit in the sauna and tell stories to each other for 30 to 45 min, that kinship is highly valuable to all of us”*.

### Transition in perceptions and behavioural meaning of home post-retirement

The experience of home was immensely related to performing everyday tasks and enjoyable activities, and these behavioural-related aspects of home changed over time. Being retired made it possible to start new projects or engage in hobbies or activities that had been neglected due to work. This could be wood work, knitting or spending more time in the garden and was perceived as enjoyable and highly appreciated. When work was no longer a part of life, the experience of home as a meaningful place changed. Before retirement, being at home contributed to health in terms of being a place to rest and gather strength for work, characterized by routines and habits. Over time, doing things at home was experienced as less stressful and there were opportunities to be creative and personal. A woman with a university degree and previously demanding work situation, retired for almost a year, said:

…before retirement I’ve seen my home like a docking station. At home I gathered strength and rested. Today I’m home all day and that (home)…means much more. When I was working I came home to charge my batteries and then I left again…

In contrast, another participant, a man who was a former interpreter, living on the countryside, expressed that he did not think the experience of home had changed so much after retirement. Still he expressed that:

I honestly don’t know how I had the time to work, it is so many things to do around the home, things that I truly enjoy.

The experience of many daily activities changed as well. Before retirement, activities at home such as making dinner and gardening were performed on routine and had concrete outcomes. Doing the same daily activities after retirement was expressed as self-rewarding and often performed simply for enjoyment and more often than before. That is, the activity repertoires were described differently; however, it was important to stay busy. The active life style originated in missing the structure provided by work which was expressed in terms of *“You have to stay busy”* or *“You cannot sit on the sofa all day”*.

### Coping with complex home ambivalence when reflecting on their future housing arrangements

The participants revealed that thinking about where to live and age is a multifaceted and individual process starting before retirement. For them, thoughts on future housing arrangements were characterized by complex home ambivalence. The mixed feelings voiced by the participants were due to health issues, identity and societal norms. The diversity of reasoning, their wishes and specific needs concerning their future housing arrangements was prominent.

The participants were aware of the normative and cultural expressions about how a younger senior should be which caused a complex home ambivalence. There was an ongoing identity negotiation such as what it means to be newly retired and how to meet societal expectations. This can be exemplified by the conflicting answers the participants gave regarding their future housing arrangements. They reflected upon their present home with warmth and they did not want to move, but somewhat contradictory, rich ideas and plans were expressed. For example, during the interview a man who had lived in the same flat in the city centre for 15 years said that he and his wife wished to remain in their present home for as long as possible. Although conflicting with the first response it was common to think about relocation, he continued:

Well, I don’t know…it is only something we talked about…other retirees go to Spain or Portugal during the winter months and come back in March…it is light and warm down there during our winter. Moving there is something we might do.

The participants showed awareness of the importance of being proactive and looking at other housing options such as age-restricted senior housing facilities or rental flats. The thoughts about different living arrangements were strikingly diverse. Some participants expressed a frustration over actors in the housing sector that produced housing options that were supposed to represent how older generations would like to live; however, they were very far from their own preferences and needs. One participant who had lived in her house situated in the countryside for five years, revealed: *“I looked around and thought that this is a home for old people, it is not a home for me”*. In her opinion, a “home for old people” symbolized powerlessness, an inactive lifestyle and not at all concurrent with the way she viewed herself.

Thoughts on relocation were related to accessibility and neighbourhood issues. There was an ongoing negotiation between “if we get frail” and wanting to “stay put”. Feeling a lack of control over one’s life situation was described as making it difficult to plan ahead. On the one hand, a possible move was dependent on health status and, on the other, on economy and the availability of appropriate housing options. Caring for a spouse and/or facing a change in accessibility due to functional decline decreased the sense of housing-related control and well-being.

## Discussion

This study explored the dynamics of perceived aspects of home and health among healthy and well-functioning younger older community-dwelling people in Sweden. The findings illustrate that home becomes progressively important after retirement, and both the immediate home environment and the local neighbourhood influence perceptions of home. Moreover, there is a complex home ambivalence as younger older people reflect on their future housing arrangements. While the findings should be interpreted in the national context of Sweden, this study makes a contribution to the existing knowledge base on home and health dynamics during the ageing process.

An important finding is that the meaning of home and former routinized daily activities change after retirement. In contrast to previous research showing difficulties in experiencing meaning of daily activities during the retirement transition (Jonsson et al. 2000), our findings suggest a more positive pattern. Many daily activities at home are seen as self-rewarding and seem to contribute to well-being. This facet of our findings is supported by previous research showing that older people who occupy their time with meaningful activities feel healthier than those who do not (Bryant et al. 2001). Furthermore, it is notable that perceptions about home become progressively important over time, among well-functioning younger older people as well as in very old age (see, for example, Iwarsson et al. 2016; Rowles and Bernard 2013). This deserves further attention and could impact planning of social services as well as the timing for housing-related counselling and interventions.

Some of the cohabiting participants living in condominiums emphasized this form of housing, where learning new skills and a variety of highly appreciated activities were offered. According to Puts et al. (2007), learning new things in old age contributes positively to well-being, and our findings show that living in condominiums or having the possibility to meet in an assembly hall could play an important role. That social aspects of home are relevant for health, aligns with our previous research showing that less social contact in the home is associated with negative health outcomes such as depression and an increase in symptoms (Haak et al. 2015; Kylén et al. 2017). Keeping in mind that loneliness is common among older people (Jennbert 2009), our findings support that it is important to provide types of housing that offer social meeting places and activities that are attractive for senior citizens (Scharlach and Lehning 2016). Housing options that senior citizens typically ask for are age-restricted housing with services such as restaurants and spaces for socializing with peers (Sandstedt and Abramsson 2012). Our findings highlight that younger older people are not a homogenous group (Lowsky et al. 2013) and contribute to the literature by reinforcing the idea that perceptions of home are neither standard nor linear. Thus, among people aged 67–70, we can expect different sets of expectations regarding housing options and housing-related interventions.

The finding that the process of planning for future housing arrangements starts already before retirement and is characterized by a complex home ambivalence aligns with findings in research on residential reasoning among very old people (Granbom et al. 2014). Relocation in old age is a major life event (Sergeant et al. 2008), a process stretched out over a long time (Nygren and Iwarsson 2009) and associated with hesitant feelings (Löfqvist et al. 2013). On the one hand, participants expressed an ongoing struggle between identity and personal preferences and, on the other, concerns about future housing arrangements. The findings imply that the societal norms of how to age successfully generate ambivalent feelings related to identity and decisions over where and how to live in the future, which in turn influence health. These beliefs can be reflected upon in the light of the successful ageing paradigm (Rowe and Kahn 1998), having both positive and negative impacts on strategies for how people relate to their personal process of ageing (Timonen 2016). Expressions such as successful, active and healthy ageing imply failure for senior citizens who do not live up to these ideals and has not yet been problematized in relation to housing.

On another note, although home is expressed as something temporary (e.g. a place of departure and return) among our younger older participants, the emotional connection to home is nevertheless evident. The emotional dimension of the meaning of home is a constantly reoccurring theme in the ageing in place literature (e.g. Dahlin-Ivanoff et al. 2007; Relph 1976; Rowles 1984; Shenk et al. 2004). Even the words used by people when describing home (comfortable, familiar, a place for rest and reflection, etc.) seem to be very similar across age cohorts and cultures (e.g. Fänge and Dahlin-Ivanoff 2009; Oswald and Wahl 2005; Wiles et al. 2012). This suggests that the emotional dimension of home is immensely related to well-being. It accentuates what has been repeatedly stated in previous research, namely that focusing solely on objective features of the home presents a limited perspective neglecting symbolic and experiential dimensions (Nygren et al. 2007). These dimensions are maybe more important to healthy and well-functioning younger older people, living in Western societies, than accessibility and other objective aspects of housing that for this group are typically not a problem.

We used previous empirical results and a the four-domain model concepts of perceived housing (Oswald et al. 2006) as a guide in the research process, not yet tested in a younger cohort. Findings support our assumption that the complexity of home needs to be broadly addressed. It is evident that home is considered in a much broader sense than only a dwelling. As noted previously (e.g. Walker 2011), it is not only the home environment but also neighbourhood and the close community play an important role for well-being as people age. This is also reflected in place theory development. For example, the Ecological Framework of Place (EFP) put forward by Moore (2014) defines place as a “socio-physical milieu involving people, the physical setting, and the program of the place, all catalyzed by situated human activity and fully acknowledging that all four may change over time”(p. 183). In contrast to Oswald et al.’s model (2006), the EFP acknowledges that the physical setting may be at different scales such as a house/apartment or the close community/neighbourhood. The EFP might be a useful theoretical lens for future research elucidating how these spatial physical and social components relate to each other.

Asking questions about everyday activities, meaningful objects or important features of home in the future was a fruitful way to elicit reflections on perceived aspects of home and health. The participants were healthy, consisted of both men and women, all living together with a partner. Taken that the majority of housing research within gerontology has focused on very old and/or single living women (e.g. Barry et al. 2017; Dahlin-Ivanoff et al. 2007; Iwarsson et al. 2016; Leith 2006), this study adds to the current knowledge base regarding home and health dynamics at different stages of the ageing process.

As to the age of the participants, the aim was to study a group of younger older people, which we have done in our previous quantitative survey studies (Haak et al. 2015; Kylén et al. 2017). For our studies, we had the possibility to access a selection from a large Swedish study/database SNAC/GÅS (See Kylén et al. 2014). The available cohort for us to access in these quantitative survey studies was 67–70 years, why also the participants in this study. The small study sample (*N* = 13) can be seen as a limitation. However, in order to validate our previous quantitative research findings and in-depth explore perceived aspects of home, interviews with a small number of participants can be an effective approach (Creswell and Plano Clark 2011). To enhance trustworthiness, site triangulation (Shenton 2004) was considered important. The participants were recruited from two different senior organizations, reflecting different socio-economic situations. Credibility was further established through carefully selecting participants from different geographical areas, and diversity in terms of gender and type of housing and by describing this process in detail.

As to the analysis process, in order to avoid overlap between categories as well as to ensure that the findings emerged from the data, several steps were taken. Following recommendations by Lincoln and Guba (1985), all co-authors were involved in the analysis process of the qualitative data. In practical terms, this was achieved through peer debriefing meetings where definitions and coding of data were discussed and examined. Through this process, emergent hypotheses were tested to see if all authors found them reasonable which enhanced confirmability, dependability and credibility of the findings. Obviously, our findings cannot be generalized to all community-dwelling people aged 67–70 years. Still, we contribute to the broader perspective of what the home means to younger generations of healthy older people through descriptions of participants’ personal experiences and discussing the findings in relation to previous research (Lincoln and Guba 1985).

Overall, our findings are to some extent contextually bounded. Sweden is recognized as one of the best countries to grow old in (Zaidi et al. 2017) and the standard of housing is generally high. Such a favourable situation might very well influence the experiences of home. Thus, studies on perceived aspects of home and health among older adults in other cultures and contexts would make a valuable contribution to the understanding of these dynamics. The selection of healthy and active participants might have an impact on the transferability of the findings to the study population at large.

## Conclusions and implications

Consistent with our previous research, the findings from this study suggest that perceived aspects of home are important to consider when developing housing-related policies and interventions. Perhaps the most compelling insights are that the meaning of home becomes progressively important during the years following retirement, and that thoughts on future housing options are characterized by a complex home ambivalence. These findings suggest that professionals involved in housing-related counselling and interventions addressing older people should address issues related to home and health already among well-functioning younger older people and not only among those with obvious problems. Our findings also suggest that social engagement and interaction with neighbours or visitors are associated with higher well-being. Housing policies should therefore place greater emphasis on promoting social aspects of the home that facilitate social participation. Moreover, the findings suggest that local neighbourhoods and secondary home places influence perceptions about home among younger older people. It is therefore important to widen the view of “home” and include these environments when planning for ageing-friendly communities.
